# Deep networks for motor control functions

**DOI:** 10.3389/fncom.2015.00032

**Published:** 2015-03-19

**Authors:** Max Berniker, Konrad P. Kording

**Affiliations:** ^1^Department of Mechanical and Industrial Engineering, University of Illinois at ChicagoChicago, IL, USA; ^2^Department of Physical Medicine and Rehabilitation, Northwestern UniversityChicago, IL, USA

**Keywords:** optimal control, deep learning, neural networks, arm reaches, motor control, motor learning

## Abstract

The motor system generates time-varying commands to move our limbs and body. Conventional descriptions of motor control and learning rely on dynamical representations of our body's state (forward and inverse models), and control policies that must be integrated forward to generate feedforward time-varying commands; thus these are representations across space, but not time. Here we examine a new approach that directly represents both time-varying commands and the resulting state trajectories with a function; a representation across space and time. Since the output of this function includes time, it necessarily requires more parameters than a typical dynamical model. To avoid the problems of local minima these extra parameters introduce, we exploit recent advances in machine learning to build our function using a stacked autoencoder, or deep network. With initial and target states as inputs, this deep network can be trained to output an accurate temporal profile of the optimal command and state trajectory for a point-to-point reach of a non-linear limb model, even when influenced by varying force fields. In a manner that mirrors motor babble, the network can also teach itself to learn through trial and error. Lastly, we demonstrate how this network can learn to optimize a cost objective. This functional approach to motor control is a sharp departure from the standard dynamical approach, and may offer new insights into the neural implementation of motor control.

## Introduction

**Figure 1:**
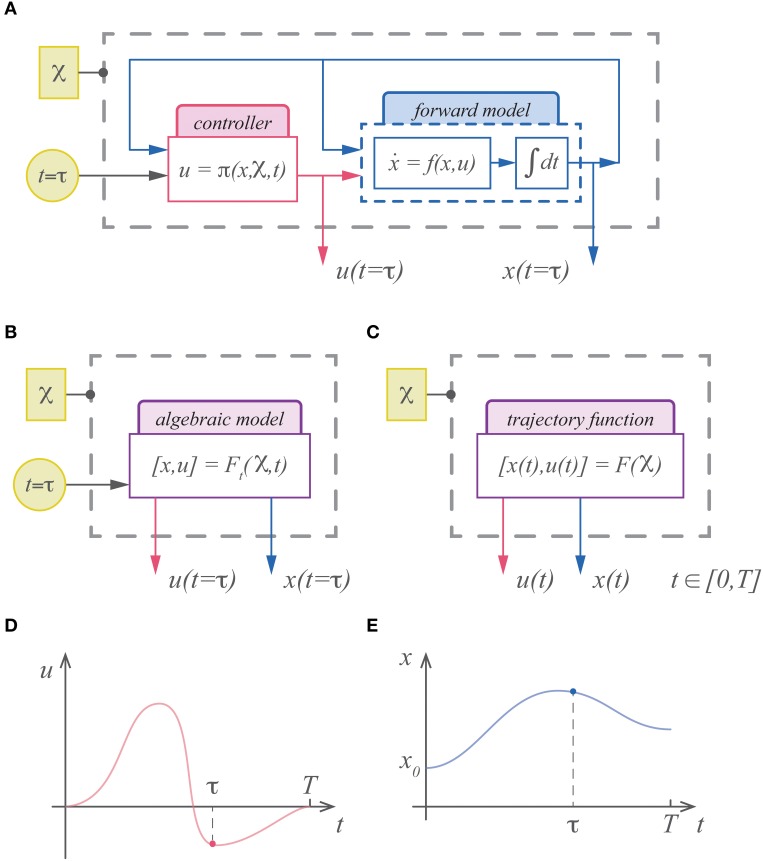
**(A)** The standard description of motor control is dynamical and must be integrated forward in time to generate commands and state estimates. **(B)** An alternative is to represent the (integrated) solution for the system. In this case the system is represented with an algebraic model that is a function of the cost parameters, boundary conditions, etc…(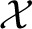) and time. **(C)** In theory the output of this model can include time as well, rendering the model an infinite dimensional trajectory function. While the standard description can only represent the command **(D)** and state **(E)** at a specific instant in time, a trajectory function represents the continuum of commands and states as its output.

That standard framework for describing the motor system is dynamical. Forward and inverse models along with a control policy represent the motor system at a specific instant in time; thus they are representations across space, but not time. To generate feedforward commands and estimated state trajectories these representations must be integrated forward in time (Figure 1A). This dynamical approach is sensible given Newton's Laws of motion and the standard descriptions of optimality (e.g., Euler-Lagrange or Hamilton-Jacobi-Bellman equations). The easy analogies between the motor system and robotics have long fueled synergies between these fields, further strengthening the dominance of these dynamical concepts. Control theory, however, informs us that there are alternatives to the dynamical description for controllers. The nervous system could therefore rely on different representations, perhaps explicitly time-varying representations of commands and trajectories.

The solution to an optimal control problem, an extremal trajectory, can be uniquely specified by the same parameters that define the control problem; e.g., the objective function, initial conditions, terminal penalties, etc… These optimal trajectories can therefore be represented with a function of time (Figure 1B). For example, with linear dynamics and quadratic costs, the optimal solution is the well-known LQR solution, which in theory could be integrated and expressed as a function of time. With even easier optimization problems such as the minimum jerk trajectory, this has already been demonstrated, and a point along the minimum jerk is described as a function of time (Hogan, 1984; Hogan et al., 1987). In theory we could express the solution as a function that maps the initial state, final state and movement duration to the (infinite dimensional) trajectory (Figure 1C). In general finding analytic expressions for these trajectory functions is intractable. Approximating these functions, however, may be relatively easy.

Recent advances in deep neural networks have appealing characteristics for approximating this optimal trajectory function. Deep architectures excel at finding hidden, low-dimensional features by discovering statistical regularities in high-dimensional training data, and can do so in a relatively unsupervised fashion (Hinton and Salakhutdinov, 2006; Bengio et al., 2007; Vincent et al., 2008, 2010; Goodfellow et al., 2009; Larochelle et al., 2009). In the context of our optimal control function, we can treat extremal trajectories as data by vectorizing both the optimal state and command (either by binning the data across time or by parameterizing the trajectories with temporal bases). To approximate these optimal trajectory functions, we have a deep network first obtain low-dimensional features, and then learn a mapping between them and the trajectories' uniquely defining parameters. This one-to-many function can be learned relatively easy through the unsupervised methods of training stacked autoencoders, and can then be refined.

Here we demonstrate that a deep network can accurately approximate optimal trajectory functions for motor control. This network maps a behavior's defining variables, such as the initial and desired final state, to the optimal outputs: a complete vectorized profile of the state and command; thus providing the roles of both a forward model and a controller. Using point-to-point reaches for data, the network is trained to simultaneously represent optimal trajectories for freely moving reaches, and those made in either a clockwise or counterclockwise curl field. We then show that this network architecture can be boot-strapped, and teach itself in a manner analogous to motor babble (Meltzoff and Moore, 1997; Dearden and Demiris, 2005; Saegusa et al., 2009). The function learned this way, though not optimal, does correctly move the limb through the desired starting and stopping locations. Finally, we demonstrate how the network can then be trained to output optimal trajectories that minimize a cost function. This functional approach to motor control is a break from traditional dynamical approaches, and offers a new framework for examining both computational and neural processes of motor control.

## Materials and methods

### Optimal trajectory functions

Here we reframe the conventional optimal control problem from one of solving a set of dynamical constraints using rate equations, to one of approximating a function. Consider a dynamical system we wish to control defined by the following rate equations,

(1) $$\dot{x}=f\left(x,\;u,\;t\right)$$

where *x* ∈ ℝ^*n*^ and *u* ∈ ℝ^*r*^ are the state and command. The cost function to be minimized is,

(2) $$J=\varphi \left(x\left(t=T\right),\;x_{d},\;T\right)\;+\;\int_{0}^{T}L\left(x\left(t\right),\;u\left(t\right),\;t\right)dt$$

where *x_d_* is the desired final state, *T* is the movement duration, *L* is the instantaneous cost associated with being in state *x* and using command, *u*, and φ is the terminal penalty. The solution is found by obtaining the optimal policy,

(3) $$u^{o}=\pi (x,\;t)$$

such that there is some corresponding state and command trajectory {*x^o^* (*t*), *u^o^*(*t*)} with *x* (*t* = 0) = *x_o_* and *t* ∈ [0, *T*] that minimizes *J*. Depending on the dynamics and the objective function, these criteria may admit multiple optimal solutions. In this study, however, we assume we can limit these solutions to one unique pair. For example, with the limb we are only interested in solutions that include counterclockwise rotations of the elbow (i.e., we ignore non-physiological solutions). Therefore, each control problem we consider has a unique optimal trajectory.

Usually these optimal trajectories are characterized by necessary and sufficient conditions for optimality, e.g., the Euler-Lagrange equations, the Hamilton-Jacobi-Bellman equations or the Pontryagin minimization principle (e.g., see Bryson and Ho, 1975; Stengel, 1994; Bertsekas, 1995). These conditions explicitly rely on the dynamical description of the system. However, as stated above, the problem description as defined by Equations (1), (2), along with the states *x_d_* and *x*_0_, are uniquely associated with the optimal trajectory. In principle therefore, some function exists that maps these parameters to the optimal solutions (Figures 1B,C),

(4) $$\left\{\varphi ,\;L,\;T,\;x_{d},\;x_{o}\right\}\to \left\{x^{o}\left(t\right),\;u^{o}\left(t\right)\right\}$$

Since we know that these solutions are parameterized by a small number of variables, they must reside in a low-dimensional manifold. Thus, we can employ standard methods to identify this low-dimensional space and approximate the optimal control functions.

Note that since time is continuous, the output of our optimal control function is infinite dimensional (Figures 1D,E). We can alleviate this difficulty by decomposing the state and command trajectories into a set of finite parameters (e.g., Fourier series coefficients, or wavelet parameters). In this study we take a straight-forward approach and directly discretize the trajectories by sampling them evenly across time at *N* points,

(5) $$\begin{array}{l} \;\left\{x^{o}\left(t\right),\;u^{o}\left(t\right)\right\}\to {\left[X^{o},\;U^{o}\right]}^{T}\\ X^{o}={\left[x^{o}\left(t_{1}\right),\;x^{o}\left(t_{2}\right),\ldots ,x^{o}(t_{N})\right]}^{T}\\ U^{o}={\left[u^{o}\left(t_{1}\right),\;u^{o}\left(t_{2}\right),\ldots ,u^{o}(t_{N})\right]}^{T} \end{array}$$

where *t_i_* = (*i*−1)*T*/(*n*−1). With this “vectorized” representation of the optimal trajectories, the output of our optimal function is now *(n + r) N*-dimensional.

Turning our attention to the function inputs, we note that for many behaviors, the objective function of Equation (2) is largely invariant. That is, though the goals for various behaviors may change in terms of what states the system is trying to visit (*x_d_*) the functional form of the underlying costs, e.g., penalties on metabolic energy, kinematics, kinetics, etc…, do not change. As such we can assume the functions φ and *L* are constant, and for our purposes implicit. For this exploratory study, we shall further restrict our attention to movements with the same movement duration, *T*. Finally, for some of our results we will include reaches in a force field, for these results we append the input with the variable, ξ, to encode the force field conditions: clockwise, counterclockwise or not present. Thus, our optimal function is of the form,

(6) $${\left[X^{o},\;U^{o}\right]}^{T}=F(x_{d},\;x_{o},\;\xi )$$

or, using a short-hand notation,

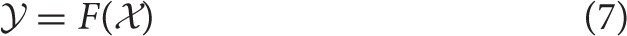



where 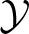 is the output (the vectorized trajectory) and 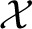 is the input. Thus, we have reframed the conventional optimal control problem from one of solving a set of dynamical constraints using rate equations, to one of approximating a function. To be clear, by no means does this imply that this reframed problem has been simplified. It is merely a different formalization of the same fundamental problem: finding the state and command trajectories that minimize a cost (Equation 2).

#### Network approximation

Though the output of our optimal trajectory function approximator is high-dimensional, 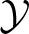 ∈ ℝ^(*n*+*r*)*N*^, we know that it is exactly a function of the low-dimensional variable, 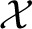 ∈ ℝ^*k*^, where *k* ≪ *N*. Thus, our optimal trajectories reside in *k*-dimensional manifold. Traditional linear methods such as principle component analysis (PCA) will fail at accurately identifying a non-linear, low-dimensional space. Deep neural networks can discover such low-dimensional representations, and can do so in an unsupervised manner. To approximate our optimal function, we construct a network in two stages. First we learn a deep autoencoder that finds a set of low-dimensional features that accurately characterize our function's outputs. Then we learn a shallow network that maps from the function's inputs to these low-dimensional features.

**Figure 2:**
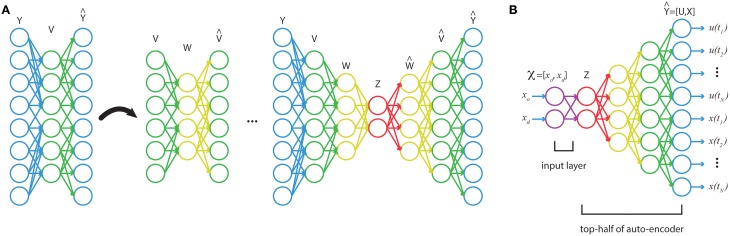
**(A)** A stacked autoencoder is trained on high-dimensional data, {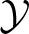
^**i**^}^**m**^**_i = 1_**, finding a series of increasingly lower-dimensional features in the data. **(B)** A shallow network trains on the map from function inputs, 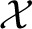 to low-dimensional features, *z*. This network is then coupled to the top-half of the stacked autoencoder to construct a function from 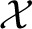, the boundary conditions, to 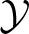 the discretized trajectory.

With a training set of optimal trajectories, {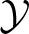
^*i*^}*^m^*_*i* = 1_, a series of feedforward autoencoders are trained, then unrolled and stacked (see Figure 2). The end result is a network that maps from the full-dimensional output, to a series of increasingly lower-dimensional representations, and then back up to a full-dimensional approximation to the output (Figure 2A). This technique has been shown to learn good low-dimensional features that accurately characterize data (Bengio et al., 2007; Vincent et al., 2008, 2010; Goodfellow et al., 2009; Larochelle et al., 2009). Using the stacked autoencoder, the optimal trajectories are mapped down to the inner-most, lowest-dimensional feature set, *z*. Then a shallow network is trained on the input-output pairs {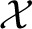
^*i*^, *z^i^*}^*m*^_*i* = 1_ via supervised learning. In the final step, the shallow network is coupled to the top-half of the stacked autoencoders (Figure 2B). The final network will have many hidden layers, which would normally make training susceptible to many poor local minima. However, the two-stage training of unsupervised, generative pretraining (for obtaining low-dimensional features) and supervised training on a shallow network (for the input layers) should yield good network weights (in the sense of small reconstruction error). Afterwards the network can be refined through standard supervised learning (see below).

### Simulations and training data

Reaches were simulated using a two-link arm model with four states: should and elbow angles (θ_*s*_, θ_*e*_) and their velocities. Realistic human limb link lengths and inertial parameters were used (obtained from Berniker and Kording, 2008, 2011). The force field was defined in terms of joint coordinates, τ = [0 *c*; ; −*c* 0] [$\dot{\theta }$_*s*_,$\dot{\theta }$_*e*_]^*T*^ where *c* was 5 Ns/rad for the clockwise field, and -5 Ns/rad for the counterclockwise field. Since we do not consider inverted elbow angles, we can define the limb's state in terms of the endpoint and its velocity in Cartesian coordinates, *x* = [*x_e_, y_e_*, $\dot{x}$_*e*_, $\dot{y}$_*e*_]^*T*^. The shoulder was defined as the origin (*x_e_* = *y_e_* = 0). Only reaches within a rectangular workspace, [−0.25, 0.25] in the *x*-direction, and [0.15, 0.55] in the *y*-direction, were considered.

Training data were optimal under Equation (2). The terminal cost was the squared Euclidean error from the target, φ = 1/2(*x*(*T*) − *x_d_*)^*T*^Φ (*x*(*T*) − *x_d_*), where Φ = 100 and *T* = 1.0. The instantaneous cost was the squared norm of the command, *L* = 1/2*u^T^*Ru, where *R* = *I*. Initial positions for the limb's endpoint were sampled uniformly over the rectangular workspace. The final desired position was obtained by choosing a point in a random direction and distance (between 1 and 15 cm) from the initial position, such that it remained within the rectangular workspace. Initial and final desired velocities were always set to zero. The resulting two-point boundary value Euler-Lagrange equations were solved in Matlab to obtain a training set of optimal trajectories {*x^io^*(*t*), *u^io^*(*t*)}^*m*^_*i* = 1_.

The corresponding unique identifiers for each reach were the initial and final desired state, (*x_d_, x_o_*), as well as a flag for the force field conditions ξ, making nine variables in total. Since all reaches started and ended with zero velocity, the inputs were reduced to a 5-element vector 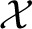
^*i*^, the initial and final desired positions as well as the field flag. All inputs and outputs were scaled to lie between zero and one to be consistent with the networks range of values. ξ encoded the force field condition with one of three values, [0.05, 0.5, 0.95].

### Training the optimal trajectory function

To train the network on optimal trajectories, 2000 random reaches under each of the three force field conditions were obtained, randomly assigning some for training (*m* = 5000), and others for validation (1000). In addition, a set of 24 test reaches were computed, for comparing the network's results across different conditions and training paradigms. Each reach's state and command trajectory was evenly sampled such that it produced a data vector, 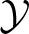
^*i*^ with (*n* + *r*)*N* = 606 elements (*dt* = 0.01). The corresponding input for each reach was the 5-element vector 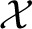
^*i*^.

Initial tests of the depth and number of nodes of the network found that relatively good reconstruction could be obtained with an autoencoder that mapped the 606-dimensional optimal trajectories down to 100 and then five hidden features (and then back up to 100, then 606 dimensions). However, to account for inaccuracies in the shallow networks map from inputs to these low-dimensional features, the size of the inner feature set, *z*, was increased to 10 for the results shown here. Therefore, the autoencoder was built with five layers. An additional two layers were used to learn the map from function inputs X to the low dimensional feature set, *z*.

The network nodes used sigmoidal activations, *f*(*h^l^_i_*) = 1/(1 + exp(*h^l^_i_*)), where *h^l^_i_* is the *i^th^* node's input in layer *l*, and *h^l^* = *W*^*l*−1^*A*^*l*−1^ + *B*^*l*−1^. *W*^*l*−1^ is the matrix of weights that connects the previous layers activations, *A*^*l*−1^ to layer *l*, and *B*^*l*−1^ is a vector of biases for the units in layer *l*. The input to the network's first layer is defined as *A*^0^ = X, and the output of the network is Y. Using software written for this purpose along with third party optimizing software (fmin), the network was trained until convergence criteria were met; either 2000 iterations of optimizing, or an error threshold was met.

### Training on self-generated data

The self-trained network used the same architecture described above, only now the input, X, was 4-dimensional since we did not examine reaches in a force field. The initial training set consisted of randomly generated reaches (*m* = 4000), made by issuing a sum of sinusoids of small random amplitude torques. These small commands to the limb resulted in reaches of relatively small displacements. With these randomly generated commands multiple reaches might start and stop at the same states but use very different commands. Thus, this first training set cannot be accurately learned with a function. However, by training on this data the network learns a consistent set of commands and trajectories that can best approximate the data in a functional form. After training, the network was used to generate a second set of training reaches by issuing random inputs to the network. The resulting set of self-generated reaches were of relatively larger displacements, but again did not land on the desired target. This process was repeated once more, producing training reaches that were adequately close to their desired final state.

### Optimizing the trajectory function

In a final exercise the network learned to optimize reaches after being self-trained. To do this we adapted the network through supervised training on the cost function (Equation 2). To do so we must compute the appropriate derivatives:

(8) $$\frac{\partial J}{\partial \theta }\;=\;\left[\frac{\partial J}{\partial X}\frac{\partial X}{\partial U}\text{ + }\frac{\partial \text{J}}{\partial \text{U}}\right]\frac{\partial \text{U}}{\partial \theta }$$

where θ is a vector of network weights. With our current architecture, we cannot compute ∂*X*/∂*U*, since the network has not learned this causal relationship, the forward dynamics of an entire trajectory. Therefore, we alter the network so that the output is now only the vectorized command, 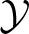 = *U*. Then a new shallow, single hidden layer network trained on the vectorized forward dynamics, that is, a mapping from the vectorized commands and the initial state, [*U, x*(*t*_1_)], to X. Note that this relationship is independent of whether or not the network is trained, or the trajectories are optimal, it is merely the input-output trajectories of the limb model. Using this forward dynamics network the deep network was optimized using gradient descent in a series of iterative steps: train the deep network using the current forward dynamics network, then train the forward dynamics network on trajectories obtained using the deep network.

An accurate and precise forward model was needed for the gradient, ∂*X*/∂*U*. However, the dynamics of the limb, under the control of our network, are necessarily low-dimensional. Therefore, the training data for learning this forward model would be impoverished, and the corresponding gradient information would be severely limited. To alleviate this complication, on each round of training/optimizing a random 50 training commands were obtained from the deep network. Random additive noise was used to make 20 unique versions of each of these commands (1000 random commands). These were combined with the randomized commands of the previous two rounds of training for a total of 3000 command trajectories. These commands and their accompanying state trajectories were then used to train the forward dynamics network. On the subsequent round a new 50 training commands were obtained and the process repeated (again using 3000 command-trajectory pairs).

Just as in the previous section, the deep network was trained on self-generated data. Once trained, the deep network produced reaches essentially identical to those displayed in the previous section. Then the network was optimized as described above. Training was halted after 300 rounds of optimizing. To overcome some of the additional computational complexities accompany this training, we down sampled the trajectories, increasing *dt* from 0.01 to 0.02. This reduced the number of parameters and sped up training and optimizing.

### PCA function

To serve as a point for comparison, a linear low-dimensional function was created using a PCA decomposition of the training data. The first five principle components were used to find a 5-dimensional feature vector, *z*, and then a linear fit was obtained between these features and the 5-dimensional input vector, 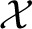. Including more components in the feature vector cannot improve performance since the input was only 5-dimensional.

## Results

We examined the ability of a deep network to represent a trajectory function; that is, a function that outputs the entire state and command trajectory for a movement. Using this network, we present results on how it can approximate reaches after training on optimal example data, train itself with self-generated data, and finally, learn to make reaches that optimize a cost function.

### A point-to-point optimal trajectory function

A deep network that approximates an optimal trajectory function was trained on point-to-point reaches moving freely through space, in a clockwise curl field, or counterclockwise curl field (defined through joint velocities, see Materials and Methods). We quantify performance with RMS errors between the approximate commands and states and their optimal counterparts. The state and commands are scaled to lie between zero and one (to be compatible with the deep network's range of outputs), so 1.0 is the maximum possible error. We complement these approximation errors with the subsequent errors that arise when the network is used as a controller. That is, small errors in the network's output, 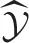 = [$\hat{U}$, $\hat{X}$], may ultimately produce large errors when using this function's command. Therefore, the predicted state may be similar to the optimal state, yet the actual state obtained may be wildly different from the optimal state trajectory. Therefore, we also compute Euclidean errors between each reach's target and the reaches terminal location when using the approximated optimal commands.

**Figure 3:**
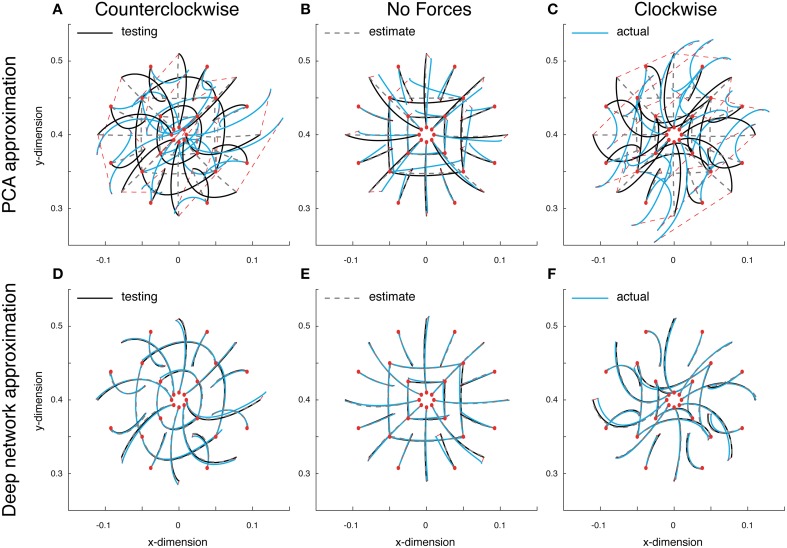
**Approximate optimal trajectory function test results**. Displayed are the optimal reaches (black lines), estimated reaches (gray dashed lines) and the actual reaches resulting from the function's outputted command (blue reaches). All reaches start at the red circles and the red dashed lines display the endpoint error. Test reaches made in a counter-clockwise curl field **(A)**, a null field **(B)** and a clockwise curl field **(C)**, using PCA model. **(D–F)** The deep networks results under the same conditions.

Since the dynamics of our system are non-linear, linear methods for representing low-dimensional features such as PCA will contain unavoidable errors. To quantify these errors as well as obtain a measure for comparison with our deep network, we first approximated the optimal trajectory function using PCA. The training error for this linear approximation was 0.037, while the validation error was 0.039. A set of test reaches performed with this PCA function approximation put these errors in perspective. Errors in the counterclockwise, null and clockwise fields on these test reaches were 0.042, 0.009, and 0.044, respectively (Figures 3A,B,C). As can be seen, the state estimates did a poor job of capturing the variations in reach curvature across force fields, and always estimated an approximately straight reach (Figures 3A,B,C, gray dashed lines). The commands too, were relatively poor (data not shown). The reaches found when using these commands were far off target, especially in the force fields, and further from optimal than the estimated reaches (Figures 3A,B,C, blue lines). The average Euclidean error between the actual reach and the target location was 6.941, 1.205, and 6.941 cm, respectively. Including more components did not help, since the 5-dimensional input invariably constrained performance. Therefore, even though our system's dynamics are low-dimensional, non-linear approaches such as our trajectory function are needed to properly describe the ongoing dynamics.

Using the same data, a deep network was trained to approximate the optimal trajectory function (see Materials and Methods for details). The training and validation error for the deep network approximation were 0.004, an order of magnitude lower than with PCA. The test reaches demonstrate obvious improvements; errors in the counterclockwise, null and clockwise fields were all 0.002. In contrast with PCA, the state estimates were close to optimal (Figures 3D,E,F, gray dashed lines), and the command errors were small enough that the resulting reaches lied on top of both the estimates and the optimal trajectories (blue lines). Here the average Euclidean errors between the actual reaches and the target locations were much improved, 0.404, 0.347, and 0.305 cm, respectively. These results offer a proof of concept that rather than use a dynamical representation for generating commands and state estimates, a function can directly represent both control and state.

### A self-trained point-to-point trajectory function

An interesting feature of using a trajectory function for control is that the network can teach itself to estimate both state and command trajectories. In a manner analogous to the learning proposed in motor babble (Meltzoff and Moore, 1997; Dearden and Demiris, 2005; Saegusa et al., 2009), example reaches generated by the untrained network, can be used to boot-strap the network. In an untrained network, randomly generated inputs 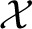 (initial and final states) will result in outputs, 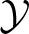, which can be used to drive the limb. Since these outputs (commands and state estimates) are untrained, using this command to drive the limb will result in a state trajectory that differs from the network's state estimate. Nonetheless, these randomly generated reaches constitute a data set to train on, by collating the commands and resulting state trajectories 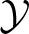
′. The correct corresponding inputs can be obtained by grouping the actual initial and final states that these random reaches gave rise to, 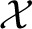
′. Thus, an untrained network can generate viable training data for itself to learn.

**Figure 4:**
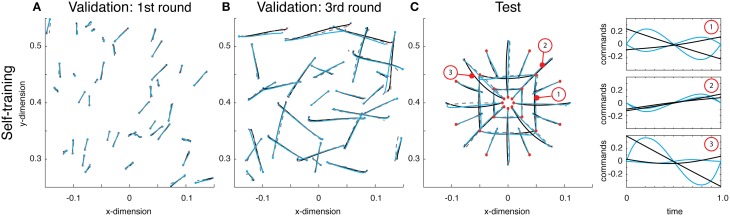
**(A)** Examples from the initial round of training's validation data, wherein reaches were generated using random small amplitude sinusoidal commands. These small torques resulted in small displacements. Displayed are the self-generated training reaches (black lines), estimated reaches (gray dashed lines) and the actual reaches resulting from the function's outputted command (blue reaches). **(B)** Validation data on the third round of training show that the reaches are very close to the desired target state. **(C)** Reaches on the test data demonstrate the network has taught itself to reach to the desired target, but does so with commands that are very different from what is optimal (see right panel for examples).

Using this idea, a network was self-trained to make point-to-point reaches. Using the same architecture as above, this network boot-strapped itself. The initial round of reaches were chosen uniformly over the entire workspace, the resulting reaches were of relatively small displacements (see Figure 4A). After training on these random reaches, however, the network was used to generate a second set of training reaches. These reaches, having already been trained, albeit on impoverished data, created relatively larger reaches. This process was repeated again, and on the third attempt, the training reaches were adequately close to their desired final state (see Figure 4B).

After training on the 3rd round of data, both the training and validation errors were 0.006. The networks commands were not optimal under Equation (2). However, they successfully brought the limb to the desired targets and the state estimates accurately predicted the trajectories. This discrepancy between optimality and these self-learned trajectories was apparent on test reaches. Here, the commands generated by the network, based on self-generated data, differed significantly from the optimal ones (see Figure 4C). The resulting error from optimal was 0.030, an order of magnitude larger than the validation error. Regardless of this deviation from optimality, the function generated commands that brought the limb to the correct target, and the average Euclidean error was 0.357 cm (within the range of errors found above when training on optimal data). Overall, these results demonstrate that a trajectory function can teach itself to generate commands and learn to estimate the resulting state trajectories.

### Optimizing the point-to-point trajectory function

We have shown that the deep network architecture can easily learn to approximate the optimal trajectory function when provided with samples, and can also teach itself to represent point-to-point reaching trajectories. In a final exercise, we demonstrate how the network can also be optimized to produce trajectories that minimize a given cost without being given the appropriate training data.

**Figure 5:**
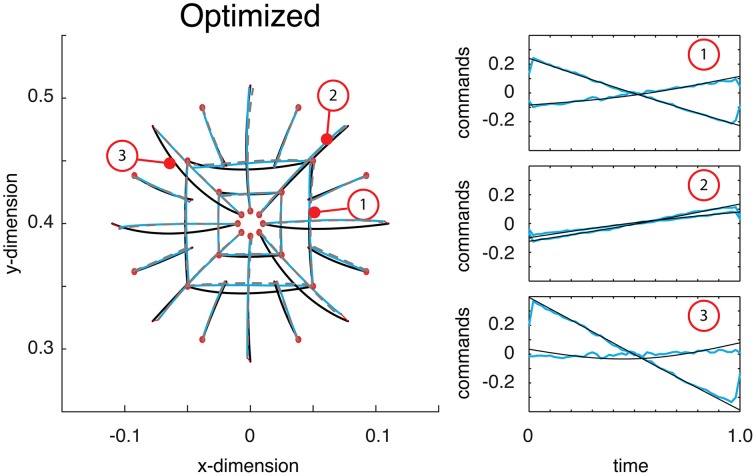
**Reaches to the test targets after being optimized**. Displayed are the optimal reaches (black lines), estimated reaches (gray dashed lines) and the actual reaches resulting from the function's outputted command (blue reaches). After being optimized, the commands are much closer to what is optimal (see right panel for examples).

Just as in the previous section, a deep network was trained on self-generated data. Once trained, the deep network produced reaches essentially identical to those displayed in the previous section. Then the network was optimized (see Materials and Methods). Comparing the function's trajectories on the test reaches the error from optimality was 0.018 (half that from above). As can be seen, the motor commands were far closer to optimal relative to their previous, self-trained values (compare Figure 4C with Figure 5). Additionally, the estimate of the reaches is relatively accurate and largely coincides with the optimal reaches (compare gray and blue lines). Throughout this optimization process, the motor commands changed dramatically, but always in such a way as to land the limb on target. The average Euclidean errors in the target locations were 0.339 cm, again very similar to previous errors. This final exercise demonstrated how a trajectory function built with a deep network can be optimized to output commands and state estimates that minimize a controller's cost function.

## Discussion

In the standard approach to motor control, state estimates and motor commands are produced using dynamical models; representations across space. Here we propose a new approach using a function to output optimal trajectories, containing both state estimates and commands for an entire reach; a representation across both space and time. We have shown how recent advances in training deep networks, in largely unsupervised ways, allow for accurate approximations to this optimal function. The resulting network can be trained to accurately represent optimal trajectories, or teach itself with self-generated data. What's more, the network can be optimized for a new cost, outputting the entire command and estimated state of an optimal reach.

The trajectory function approach has some obvious weaknesses. Using a deep network requires a large number of parameters, and in turn a lot of training data for successful learning. Similarly, simultaneously representing states and commands across time also increases the number of parameters and required training data. For our example two-link limb this data was easily obtained, but in other contexts, e.g., high-dimensional systems where computing optimal trajectories is computationally intensive, this may be impractical. Yet, without a formal method for approximating the analytical solutions to these optimal control functions, using network approximations such as these may be the second best option.

With regard to the nervous system, simulating large data sets for training may be neither feasible, nor necessary. The nervous system has at its disposal a very good means of generating large data sets of motor information, its own body. A long-standing hypothesis regarding the early stages of motor learning is that seemingly random motor commands and their consequences, termed motor babble, may be used to train-up the motor system. While this idea has been useful in framing motor learning for both biological and robotic systems (Meltzoff and Moore, 1997; Dearden and Demiris, 2005; Saegusa et al., 2009), it only facilitates the learning of a forward model (by relating inputs to outputs). It is not obvious how such random commands can be used to train a control policy. In the approach we present, any command and state trajectory pair are a valid training sample. This fact allows the trajectory function to boot-strap itself in a manner consistent with motor babbling.

Another potential weakness is the fact that our trajectory function produces command and state trajectories over a fixed length of time. Being that the dynamics of the limb are non-linear, the network's outputs cannot be scaled in time to produce accurate movements of longer or shorter durations. Fortunately it is easy to propose potential solutions to this difficulty. For example, movement duration could be included as an input to the trajectory function, and the same training procedures could be implemented without change. Alternatively, rather than representing commands and states across discretized time, basis functions could be used, whose width could be varied as a function of the movement time.

No doubt other possibilities exist to improve upon the discretized representation of time. However, this “bug” may in fact be a feature. For example, if the function's output modulated temporal bases, then the motor system would reflect these temporal regularities in it's commands (d'Avella et al., 2003). Similarly, submovements, the apparent building blocks of human movements, may share some features with our trajectory function's temporal properties (Miall et al., 1993; Doeringer and Hogan, 1998; Krebs et al., 1999; Fishbach et al., 2007). The existence of submovements implies reaches are generated with a sequence of finite duration commands. It is interesting to speculate how this might be the result of a trajectory function not unlike the one we propose here, constructing motor commands with a series of finite-duration signals. Future work can examine the connections between using a trajectory function and the ensuing submovements.

The trajectory function approach offers multiple benefits too. As noted above, the architecture can generate its own training data. Since the network directly represents an entire trajectory, examining global features of reaches, such as curvature, velocity profiles, and motor effort are relatively easy. Additionally, although we have implemented a feedforward network in this preliminary examination, future work could implement the same trajectory function with a probabilistic network, e.g., a deep Boltzman machine (Hinton et al., 2006; Salakhutdinov and Hinton, 2009). With such a representation, the entire function is bidirectional, and can act as both a forward and inverse model simultaneously. What's more, unlike an inverse dynamical model, an inverse trajectory function could be used to find the entire command trajectory for an arbitrary state trajectory (e.g., the command necessary to reach around an obstacle).

Being that our functional approach is a sharp departure from the conventional dynamical approach, we end by speculating on how it could be neurally implemented and what potential insight it may offer. In the conventional approach, neurons encode the dynamics of the motor system, in which case neural activity represents the instantaneous state or command of a reach. Thus, this is a centralized representation of commands and state, encoding their temporal changes through time-varying neural activity. In contrast, with the approach we present many groups of neurons are used to encode the temporal profile of states and commands at distinct points in time. To drive the motor system this information must be conveyed in a serial fashion. This could be achieved by chaining the network's outputs into an ordered sequence of activity. Thus, our functional approach is a spatially distributed representation, encoding time-varying signals through changes in the spatial location of neural activity.

Despite a long history of electrophysiological studies, it is not clear which of these two approaches best explains the evidence. While there is a lot of evidence to support the conventional approach it is largely based on aggregate neural activity (Shidara et al., 1993; Gomi et al., 1998; Kobayashi et al., 1998; Schweighofer et al., 1998a,b). Yet the activity of individual neurons is only locally associated with changes in states and commands (Coltz et al., 1999; Cohen and Andersen, 2002; Paz et al., 2003; Georgopoulos et al., 2007), arguably consistent with the spatially distributed nature of the functional approach. There is also a large body of work that demonstrates sequences of movements are encoded with parallel ensembles of activity, whose temporal ordering of activity may encode movement segment sequences (Tanji, 2001; Averbeck et al., 2002). It is easy to speculate that this same encoding of temporal sequences of movements could be recapitulated to generate the finer temporal-scale of an individual movement, a possibility that has computational and empirical evidence (Abeles et al., 1993; Hayon et al., 2005).

Finally, there is a recently renewed enthusiasm for interpreting neural activity in terms of a dynamical systems perspective (as distinct from the representation of a dynamical forward model) wherein the dynamics of a network of neurons are tuned to encode the time-varying motor information (Shenoy et al., 2013). In this framework a neuronal state's trajectory is determined by driving the system to an appropriate initial state. This is not entirely unlike delivering the appropriate input to our network, which then activates the correct output activities. Further, it is argued that the neuronal state, the aggregate activity of many neurons, drives the motor commands (Churchland et al., 2012). With our functional approach, it is not difficult to imagine how a linear combination of the sequential activity across the output nodes might be consistent with this evidence too. Although speculative at this point, predictions such as these may help to distinguish between different explanations for how the brain encodes the necessary time-varying motor commands.

### Conflict of interest statement

The authors declare that the research was conducted in the absence of any commercial or financial relationships that could be construed as a potential conflict of interest.
